# Amyloid-β Peptide Exacerbates the Memory Deficit Caused by Amyloid Precursor Protein Loss-of-Function in *Drosophila*

**DOI:** 10.1371/journal.pone.0135741

**Published:** 2015-08-14

**Authors:** Isabelle Bourdet, Aurélie Lampin-Saint-Amaux, Thomas Preat, Valérie Goguel

**Affiliations:** Genes and Dynamics of Memory Systems, Brain Plasticity Unit, CNRS, ESPCI-ParisTech, PSL Research University, 10 rue Vauquelin, 75005 Paris, France; National Center for Geriatrics and Gerontology, JAPAN

## Abstract

The amyloid precursor protein (APP) plays a central role in Alzheimer’s disease (AD). APP can undergo two exclusive proteolytic pathways: cleavage by the α-secretase initiates the non-amyloidogenic pathway while cleavage by the β-secretase initiates the amyloidogenic pathway that leads, after a second cleavage by the **γ**-secretase, to amyloid-β (Aβ) peptides that can form toxic extracellular deposits, a hallmark of AD. The initial events leading to AD are still unknown. Importantly, aside from Aβ toxicity whose molecular mechanisms remain elusive, several studies have shown that APP plays a positive role in memory, raising the possibility that APP loss-of-function may participate to AD. We previously showed that APPL, the *Drosophila* APP ortholog, is required for associative memory in young flies. In the present report, we provide the first analysis of the amyloidogenic pathway’s influence on memory in the adult. We show that transient overexpression of the β-secretase in the mushroom bodies, the center for olfactory memory, did not alter memory. In sharp contrast, β-secretase overexpression affected memory when associated with APPL partial loss-of-function. Interestingly, similar results were observed with *Drosophila* Aβ peptide. Because Aβ overexpression impaired memory only when combined to APPL partial loss-of-function, the data suggest that Aβ affects memory through the APPL pathway. Thus, memory is altered by two connected mechanisms—APPL loss-of-function and amyloid peptide toxicity—revealing in *Drosophila* a functional interaction between APPL and amyloid peptide.

## Introduction

The Amyloid Precursor Protein (APP) is a major actor of Alzheimer's disease (AD), a progressive neurodegenerative disorder in which the first symptom is the loss of episodic memory [1]. APP is a transmembrane protein that can be cleaved by membrane-associated proteases following two exclusive pathways: on the one hand, cleavage by the α-secretase initiates the non-amyloidogenic pathway that generates a large secreted fragment sAPPα; on the other hand, the amyloidogenic pathway is initiated by the β-secretase cleavage that liberates sAPPβ and, after a second cleavage by γ-secretase, extracellular Aβ peptides [2]. Aggregation of Aβ peptides forms extracellular amyloid plaques, a major histopathological hallmark of AD.

Beta-site APP Cleaving Enzyme-1 (BACE1) is the major neuronal β-secretase in the human brain [3]. BACE1 has been shown to have also many additional substrates [4]. Although genetic analyses have failed to uncover any BACE1 mutation in patients with familial hereditary AD (FAD), increased β-secretase activity has been reported in FAD [5], and increased expression has been found in cortex of sporadic AD patients [6–9]. Thus, elevated BACE1 cerebrospinal fluid levels have been proposed as an early biomarker for AD pathology. Due to its rate limiting function in Aβ production [10], BACE1 has been a prime therapeutic target to prevent Aβ generation in AD [11]. However, none of the numerous inhibitors developed that successfully decrease Aβ release helped preventing the cognitive decline [12], suggesting that Aβ accumulation might not be the only early event leading to AD.

Several studies have indicated a positive role for BACE1 in memory processes. BACE1 null mice manifest alterations in performance on cognition [13], while BACE1-mediated cleavage of APP can facilitate learning and memory [14]. In contrast, BACE1 deficiency has been shown to rescue memory deficits in mice models of AD [15,16], and more recently, it was reported that knock-in of human BACE1 leads to age-dependent deficit semantic-like memory [17].

Functional studies of the APP pathway in rodents are limited because of redundancy due to three APP-related genes and the lethality of the triple knockout [18,19]. Moreover, AD mouse studies have mainly relied on the use of constitutive mutants, so that developmental functions cannot be easily discriminated from direct roles in the adult brain. In contrast, *Drosophila melanogaster* genome encodes a single non essential APP ortholog named APP-Like (APPL) [20], and in this organism, expression of genes of interest can be controlled both spatially and temporally [21]. Although relatively simple (100,000 neurons) [22], the *Drosophila* brain is highly organized and is able to drive various sophisticated behaviors. The mushroom bodies (MB), a prominent bilateral structure of the insect brain comprising 2,000 neurons per brain hemisphere [23], constitute the olfactory learning and memory center [24]. Depending on the sequence of an aversive associative training protocol, distinct types of olfactory memory can be formed [25], and remarkably, molecular processes at play are conserved from flies to mammals [26].

APPL is enriched in the adult MB [27]. Importantly, α- β- and γ-secretases have been characterized in *Drosophila*, and APPL has been shown to undergo proteolytic pathways similar to that of APP [28]. Although human APP and *Drosophila* APPL show no sequence similarity at the level of the Aβ sequences, an Aβ-like peptide (dAβ) generated after a β-secretase initial cleavage of APPL has been recently characterized in the fly [29]. Upon aging, APPL overexpression leads to ThioflavineS-positive aggregates that are associated with neurodegeneration, suggesting that APPL processing generates a functional analog of human Aβ [29].

Transgenic flies have been generated to study human Aβ-induced toxicity [30]. Neuronal expression of secreted human Aβ42 results in many of the features observed in the mouse model, and moreover, the severity of the neurodegenerative phenotypes correlates with the amyloidogenic properties of the Aβ peptides [31–36]. Thus similarities between neurotoxic biochemical pathways induced by Aβ deposition in flies and humans indicate that *Drosophila* constitutes a relevant model to study the molecular basis of AD pathogenesis.

Several studies in rodents have suggested that APP plays a positive role in memory [37–39]. Similarly, we showed in *Drosophila* that APPL is required for memory in young adults [40,41], supporting the hypothesis that APP loss-of-function may contribute to AD initial symptoms. Here we provide the first analysis of the influence of the amyloidogenic pathway on memory in adults. We investigated the effect on olfactory memory of the transient overexpression of the *Drosophila* β-secretase, dBACE [29]. Strikingly, dBACE overexpression in the MB of young flies led to a memory defect only when associated to APPL partial loss-of-function. Furthermore, similar data were obtained when dAβ was overexpressed in the MB. The results thus suggest that memory is affected by two connected mechanisms: amyloid peptide toxicity and APPL loss-of-function.

## Materials and Methods

### Drosophila stocks

All fly strains were outcrossed to a Canton Special genetic background. *UAS-dBACE* (*dBACE*) and *UAS-dAβ (dAβ)* lines were kindly provided by D. Kretzschmar [29]. The *Appl* ^*d*^ *;MBSw* line is described in [41]. For behavioral and quantitative PCR experiments, flies were raised on standard medium with 60% humidity under a 12 h light/dark cycle at 18°C and 25°C, respectively. As the *Appl* gene is located on the X chromosome, only females were analyzed for experiments that involved *Appl* ^*d*^ genotypes. To induce transgene expression, the GeneSwitch system was used as described [42]. A stock solution of RU486 (SPI-Bio) (10 mM in 80% ethanol) was mixed into molten food at 65°C to a final concentration of 200 μM. The TARGET system [43] was induced by incubating flies at 30°C for 3 days.

### Behavior experiments

Flies were trained with classical olfactory aversive conditioning protocols as described [44]. 1–2 day old flies were kept on RU486-containing medium (RU) for 48 h prior to conditioning. Training and testing were performed at 25°C with 80% humidity. Conditioning was performed on samples of 30–40 flies with 3-octanol (>95% purity; Fluka 74878, Sigma-Aldrich) and 4-methylcyclohexanol (99% purity; Fluka 66360, Sigma-Aldrich) at 0.360 mM and 0.325 mM, respectively. Odors were diluted in paraffin oil (VWR International, Sigma Aldrich). Memory tests were performed with a T-maze apparatus [45]. For 1 min flies were allowed to choose between two arms, each delivering a distinct odor. An index was calculated as the difference between the numbers of flies in each arm divided by the sum of flies in both arms. A performance index results from the average of two reciprocal experiments with either octanol or methylcyclohexanol as conditioned stimulus. For odor avoidance tests after electric shock exposure, and response to electric shock, flies were treated as described [44].

### Quantitative PCR analyses

Total RNA was extracted from 50 female heads with the RNeasy Plant Mini Kit (Qiagen), submitted to DNase I treatment (Biolabs), and further reverse transcribed with oligo(dT)20 primers using the SuperScript III First-Strand kit (Life Technologies) according to the manufacturer’s instructions. We compared the level of the target cDNA to that of the *α-Tub84B* (CG1913) cDNA, which was used as a reference. Amplification was performed using a LightCycler 480 (Roche) in conjunction with the SYBR Green I Master (Roche). For each experiment, reactions were carried out in triplicate for two dilutions of each cDNA. The *n* represents the number of independent experiments performed. To measure specifically mRNA resulting from the *UAS-dAβ* transgene, we took advantage of the HA-tag coding sequence that has been inserted in 3’ of the dAβ sequence [29]. Specificity and size of amplification products were assessed by melting curve analyses and agarose gel electrophoresis, respectively. Expression relative to the reference is expressed as a ratio (2^-ΔCp^, where Cp is crossing point).

### Statistical analyses

Memory scores are displayed as mean ± standard error of the mean (SEM). To compare performance indexes from more than two groups, statistical analyses were performed through a 1-way ANOVA, followed by Newman-Keuls pairwise comparisons. Overall ANOVA *p*-value is given in the legends along with the value of the corresponding Fisher distribution *F*_*(x*,*y)*_, where *x* is the number of degrees of freedom for groups and *y* the total number of degrees of freedom for the distribution, while asterisks on the figure represent the pairwise *post hoc* comparisons, following the usual nomenclature. Except for graphs comprising the *Appl* ^*d*^ */+;MBSw/+* genotype, the wild-type genotype is not included in the ANOVA. To compare memory scores of two groups, Student’s *t* tests were used with *p* < 0.05 as a significance threshold. mRNA quantification measurements were analyzed from 2^-ΔCp^ ratios in the same way.

## Results

It has been suggested that deregulation of BACE1 gene expression could play an important role in AD pathogenesis [46]. Nevertheless, the incidence of the β-secretase overactivity restricted to the adult animal has never been addressed. We aimed at studying the effect on memory of β-secretase overexpression in the adult fly. We took advantage of the conditional GeneSwitch system, a genetic tool that allows both spatial [47] and temporal control of the expression of genes of interest: GeneSwitch becomes active when flies are fed with the RU486 ligand (RU, [48]).

*Drosophila* olfactory memory can be assessed using a classical aversive conditioning paradigm in which flies are successively exposed to two distinct odors, one of which is paired with pulses of electric shock. Depending on the sequence of the training, flies form distinct memory phases [25]. Learning is assessed immediately after a single cycle of conditioning, whereas short-term memory (STM) is assessed 2 h after.

**Fig 1:**
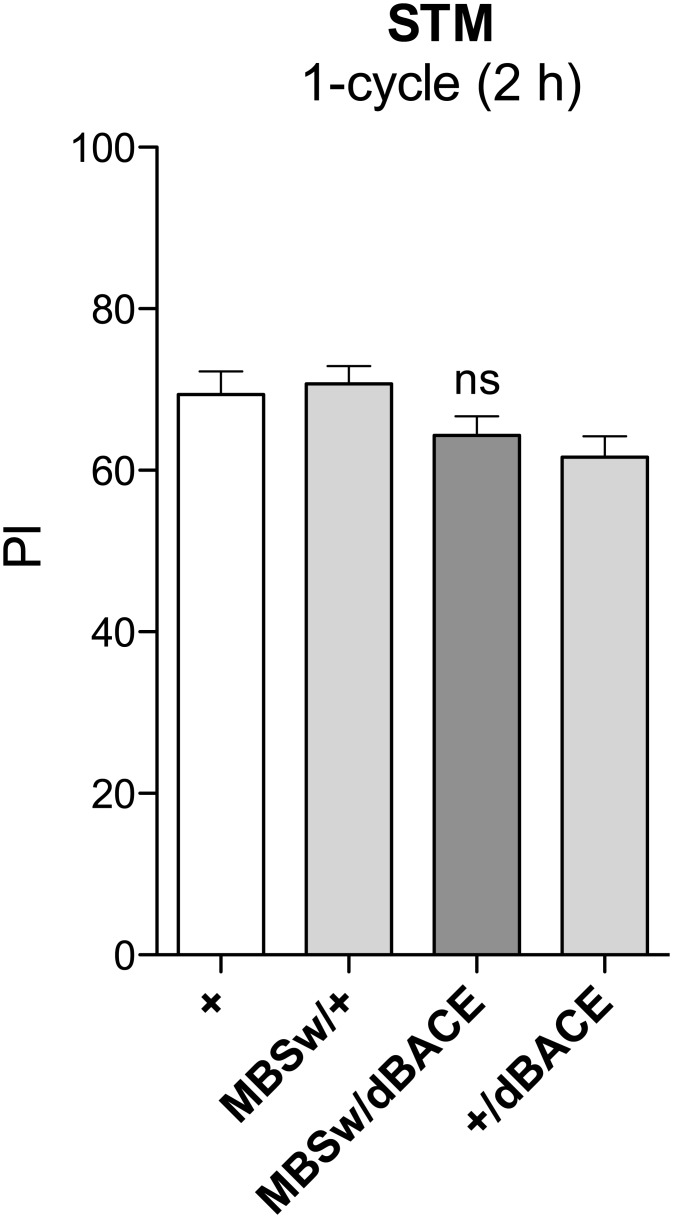
dBACE overexpression in adult MB does not alter memory. Flies were fed with RU for 48 h before conditioning to induce *UAS-dBACE* transgene expression. STM assessed 2 h after one training session is not affected. The score of *MBSw*/*dBACE* flies is not different from that of the genetic control groups (*F*_(2,77)_ = 4.048, *p = 0.0214, *n* ≥ 24, Newman–Keuls *post-hoc*, *MBSw*/*dBACE* vs +/*dBACE p* > 0.05, *MBSw*/*dBACE* vs *MBSw*/+ *p* > 0.05). Bars, Mean ± SEM. PI, Performance index.

We analyzed consequences of dBACE overexpression in the MB using the MB247 driver that is expressed in the α/β and γ neurons [49]. To restrict expression in the adult MB, we took advantage of the MB-Switch driver (MBSw, [42]) and a *UAS-dBACE* transgene [29]. Flies overexpressing dBACE (*MBSw/dBACE*) exhibited a STM score that was not significantly different from that of control flies (Fig 1), showing that STM is not affected by dBACE overexpression in the MB of young adult flies.

To get a better insight into the potential effect of dBACE overexpression on memory in link with the APPL pathway, we analyzed the effect of dBACE overexpression in flies expressing a reduced level of its APPL target. In a previous work, we knocked down APPL expression in the adult MB by RNA interference (RNAi) with *UAS–RNAi* transgenes [40]. However, we did not wish to overexpress simultaneously dBACE and an *Appl-RNAi* construct because the presence of two *UAS*-transgenes in the fly genome may lead to a substantial decrease in the Gal4-mediated transcription of each of them. Thus, to avoid a decreased efficiency of *Appl* knock-down, we took advantage of the *Appl* ^*d*^ null mutant [50] to generate an APPL partial loss-of-function (LOF) genetic context. We recently described a *Drosophila* model that endogenously express about half the wild-type level of *Appl* and the conditional MBSw driver (*Appl* ^*d*^ */+;MBSw/+* [41]). *Appl/+;MBSw/+* heterozygous flies exhibit a normal learning while STM is affected [41]. Although the APPL partial LOF thus achieved is not conditional, we showed that the resulting STM deficit is functional as it can be rescued by overexpressing an APPL form exclusively at the adult stage [41].

**Fig 2:**
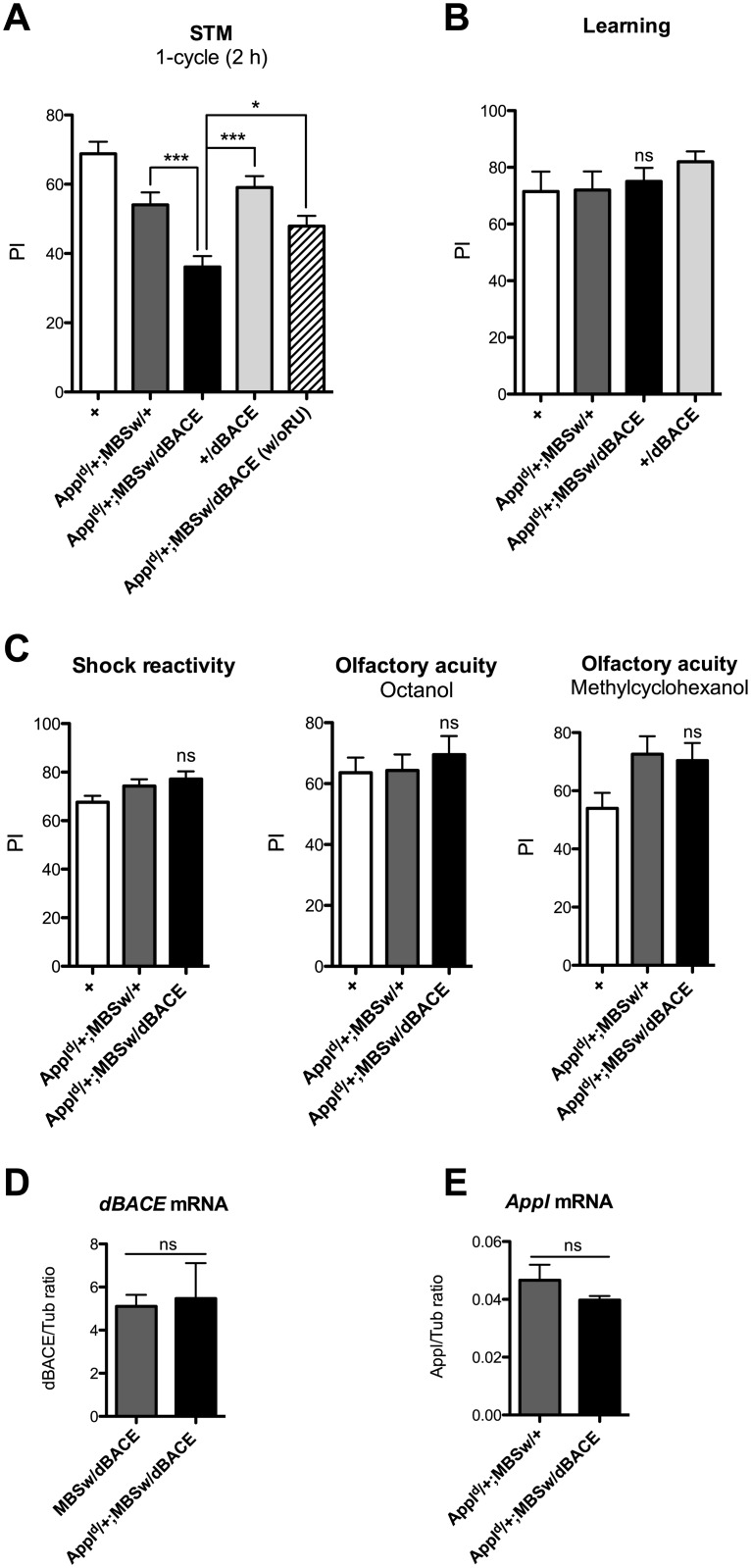
dBACE overexpression exacerbates the memory deficit caused by APPL partial loss-of-function. Unless indicated (A, w/o RU), flies were fed with RU for 48 h. (A) Flies were submitted to one cycle training and tested 2 h later. *Appl* ^*d*^ */+;MBSw/+* flies show an STM deficit (*F*_(4,101)_ = 11.99, ***p < 0.0001, *n* ≥ 14, Newman-Keuls *post-hoc*, *Appl* ^*d*^ */+;MBSw/+* vs *+* *p < 0.05), and *Appl* ^*d*^/+;*MBSw*/*dBACE* flies exhibit a STM score significantly lower than the genetic controls (Newman-Keuls *post-hoc*, *Appl* ^*d*^/+;*MBSw*/*dBACE* vs *Appl* ^*d*^ /+;MBSw/+ ***p < 0.001, *Appl* ^*d*^/+;*MBSw*/*dBACE* vs *+*/dBACE ***p < 0.001). *Appl* ^*d*^/+;*MBSw*/*dBACE* flies not fed with RU (w/o RU) display a STM score significantly different from flies of the same genotype fed with RU (Newman-Keuls *post-hoc*, *p < 0.05), and similar to *Appl* ^*d*^/+;*MBSw*/*+* flies (Newman-Keuls *post-hoc*, *p* > 0.05). (B) Learning is not affected. To assess learning, flies were tested immediately after one cycle training (*F*_(2,27)_ = 0.8522, *p* = 0.4385, *n* ≥ 8). (C) Neither shock reactivity (*F*_(2,21)_ = 2.747, *p* = 0.0896, *n* ≥ 7) nor olfactory acuity (octanol, *F*_(2,46)_ = 0.3490, *p* = 0.7073, *n* ≥ 15; methylcyclohexanol, *F*_(2,52)_ = 2.959, *p* = 0.0610, *n* ≥ 17) is impaired. Bars, Mean ± SEM. PI, Performance index. (D, E) Analysis of *dBACE* and *Appl* transcription. Total RNA was extracted from *MBSw*/*dBACE*, *Appl* ^*d*^/+;*MBSw*/+ and *Appl* ^*d*^/+;*MBSw*/*dBACE* heads. Resulting cDNA was quantified using tubulin (Tub) expression as a reference. Results shown are ratios to the reference. (D) Quantification of *dBACE* mRNA level (*t* test, *p* = 0.8376, *n* = 4). (E) Quantification of *Appl* mRNA level (*t* test, *p* = 0.2330, *n* = 3). Bars, Mean ± SEM. ns, not significant.

To assess the effect of dBACE overexpression in an APPL partial LOF genetic background, we analyzed dBACE overexpression in *Appl* ^*d*^/+;*MBSw*/+ flies. As previously described, *Appl* ^*d*^ */+;MBSw/+* flies displayed a score significantly lower than wild-type (+), showing that their STM is impaired (Fig 2A). Strikingly, *Appl* ^*d*^/+;*MBSw*/*dBACE* flies overexpressing dBACE exhibited an STM score significantly lower than their genetic controls (Fig 2A). When *Appl* ^*d*^/+;*MBSw*/*dBACE* flies were not fed with RU, they displayed a score significantly higher than that of flies of the same genotype fed with RU (Fig 2A). These data show that the observed memory decrease is RU-specific and thus due to the induction of dBACE overexpression in the adult MB in an APPL LOF context. Interestingly, learning capacity remained unaffected (Fig 2B). We also assessed whether *Appl* ^*d*^/+;*MBSw*/*dBACE* flies perceived normally the stimuli used for conditioning. Their ability to avoid electric shocks was unaffected, as was their response to each odor after electric shock exposure (Fig 2C). Altogether, the data demonstrate that in an APPL partial LOF context, dBACE overexpression is deleterious for STM. Moreover, since dBACE overexpression does not affect memory capacity of flies with wild-type level of APPL, we conclude that the memory deficit induced by dBACE overexpression is functionally related to the APPL pathway. We next analyzed the level of dBACE overexpression in wild-type and APPL LOF flies. The level of dBACE mRNA was similar in *MBSw/dBACE* and *Appl* ^*d*^/+;*MBSw*/*dBACE* fly heads (Fig 2D), indicating that the augmentation of the memory deficit observed when dBACE is overexpressed in an APPL partial LOF is not caused by an increase in the level of dBACE expression. We also analyzed whether the memory deficit induced by dBACE overexpression in *Appl* ^*d*^/+;*MBSw*/*dBACE* flies was linked to a modulation of *Appl* level of expression. *Appl* mRNA level was similar in *Appl* ^*d*^/+;*MBSw*/+ and *Appl* ^*d*^/+;*MBSw*/*dBACE* flies (Fig 2E), showing that the mechanism by which dBACE exacerbates the memory deficit is not through modulation of *Appl* transcription.

**Fig 3:**
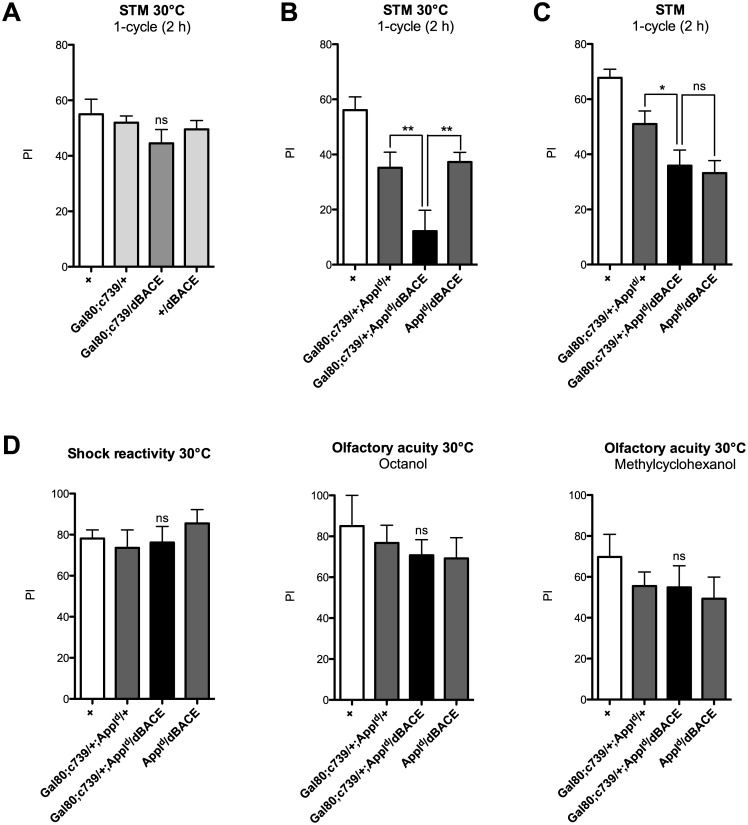
dBACE overexpression in the α/β neurons exacerbates the memory deficit caused by APPL partial loss-of-function. (A, B, D) In order to induce Gal4-dependent transcription, flies were incubated for 3 days at 30°C. (A, B, C) Flies were submitted to one-cycle training, and memory was assessed 2 h later. (A) STM is not affected when dBACE is overexpressed. The score of *Gal80;c739/dBACE* flies is not different from that of the two genetic control groups (*F*_(2,48)_ = 1.045, *p* = 0.3599, *n* ≥ 16). (B) As expected, *Gal80;c739/+;Appl* ^*d*^/+ flies display a STM deficit (*F*_(3,46)_ = 10.57, ***p < 0.0001, *n* ≥ 11, Newman-Keuls *post-hoc*, *Gal80;c739/+;Appl* ^*d*^/+ vs + *p < 0.05). *Gal80;c739/+;Appl* ^*d*^/*dBACE* flies exhibit a memory score significantly lower than their two genetic controls (Newman-Keuls *post-hoc*, *Gal80;c739/+;Appl* ^*d*^/*dBACE* vs *Gal80;c739/+;Appl* ^*d*^/+ **p < 0.01, and *Gal80;c739/+;Appl* ^*d*^/*dBACE* vs *Appl* ^*d*^/dBACE **p < 0.01). (C) When *Gal80;c739/+;Appl* ^*d*^/*dBACE* flies were not incubated at 30°C, they display a STM score that is not significantly different from one of their genetic control groups (*F*_(3,64)_ = 9.313, ***p = 0.0001, *n* ≥ 12, Newman-Keuls *post-hoc*, *Gal80;c739/+;Appl* ^*d*^/*dBACE* vs *Gal80;c739/+;Appl* ^*d*^/+ *p < 0.05, and *Gal80;c739/+;Appl* ^*d*^/*dBACE* vs *Appl* ^*d*^/*dBACE p >* 0.05). (D) Neither shock reactivity (*F*_(2,33)_ = 0.6421, *p* = 0.533, *n* ≥ 9) nor olfactory acuity (octanol, *F*_(2,31)_ = 0.1687, *p* = 0.8456, *n* ≥ 8; methylcyclohexanol, *F*_(2,31)_ = 0.1016, *p* = 0.9037, *n* ≥ 8) is impaired. Bars, Mean ± SEM. PI, Performance index.

We further analyzed the effect of dBACE overexpression using a more specific driver. C739 has been shown to label specifically the α/β neurons of the MB [51]. To restrict Gal4 expression to adulthood, we took advantage of the TARGET system that relies on a thermo-sensitive Gal80 inhibitor that becomes inactive at 30°C [43]. We observed that dBACE overexpression driven by the tub-Gal80^ts^;c739 driver (Gal80;c739), did not affect STM (Fig 3A). We next aimed to analyze dBACE overexpression in APPL partial LOF flies. As previously observed, APPL LOF flies (*Gal80;c739/+;Appl* ^*d*^/+) showed a memory score lower than wild type (Fig 3B). Furthermore, *Gal80;c739/+;Appl* ^*d*^/*dBACE* flies showed a STM score lower than their genetic control groups (Fig 3B). The memory impairment was not observed when flies were not incubated at 30°C (Fig 3C), showing that it was previously caused by dBACE induction. We verified that *Gal80;c739/+;Appl* ^*d*^/*dBACE* flies showed normal response to electric shock exposure and olfactory acuity (Fig 3D). In conclusion, these results are similar to that obtained with the MB247 driver, namely dBACE overexpression does not affect STM in normal flies, whereas it does exacerbate the memory deficit caused by APPL LOF. Furthermore, they are consistent with APPL being highly expressed in the α/β neurons [52] known to be involved in aversive 2-hour labile memory and LTM [53], the memory phases specifically affected by APPL partial loss-of-function [40,41].

**Fig 4:**
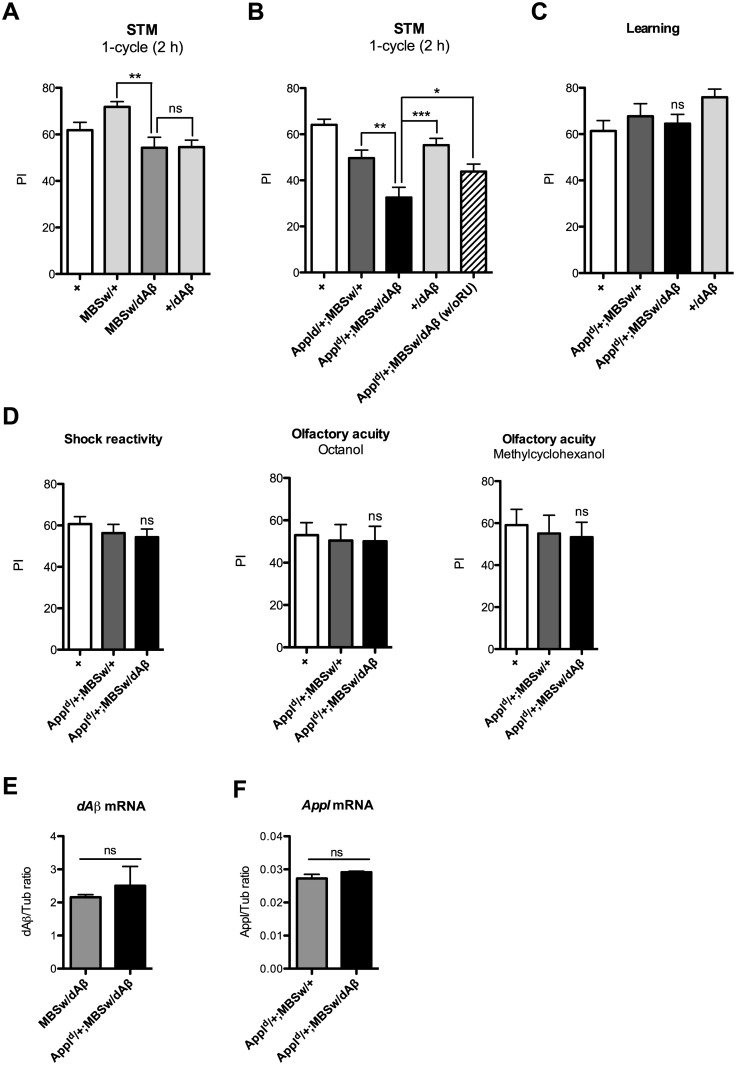
dAβ expression alters memory only when associated with APPL partial loss-of-function. Unless indicated (B, w/o RU), flies were fed with RU for 48 h prior to either conditioning or mRNA extraction. (A, B) Flies were submitted to one cycle training and memory was tested 2 h later. (A) *MBSw*/*dAβ* flies exhibit a STM score similar to one of the genetic control groups (*F*_(2,53)_ = 8.421, ***p = 0.0007, *n* ≥ 17, Newman-Keuls *post-hoc*, *MBSw*/*dAβ* vs *+*/*dAβ p* > 0.05, *MBSw*/*dAβ* vs *MBSw*/*+* **p < 0.01). (B) *Appl* ^*d*^ */+;MBSw/+* flies show an STM deficit (*F*_(4,144)_ = 12.41, ***p < 0.0001, *n* ≥ 26, Newman-Keuls *post-hoc*, *Appl* ^*d*^ */+;MBSw/+* vs *+* **p < 0.01). *Appl* ^*d*^/+;*MBSw*/*dAβ* flies display a score significantly decreased compared to the controls (Newman-Keuls *post-hoc*, *Appl* ^*d*^/+;*MBSw*/*dAβ* vs *Appl* ^*d*^ /+;MBSw/+ **p < 0.01, *Appl* ^*d*^/+; *MBSw*/*dAβ* vs *+*/dAβ ***p < 0.001). *Appl* ^*d*^/+;*MBSw*/*dAβ* flies not fed with RU (w/o RU), exhibit scores significantly higher than flies of the same genotype fed with RU (Newman-Keuls *post hoc*, *p < 0.05), and similar to that of *Appl* ^*d*^ */+;MBSw/+* control flies (Newman-Keuls *post hoc*, *p* > 0.05). (C) Learning is not affected. To assess learning, flies were submitted to one cycle training and tested immediately after (*F*_(2,30)_ = 1.684, *p* = 0.2038, *n* ≥ 9). (D) *Appl* ^*d*^/+;*MBSw*/*dAβ* flies exhibit wild-type shock reactivity (*F*_(2,34)_ = 0.7100, *p* = 0.4992, *n* ≥ 11), as well as wild-type olfactory acuity (octanol, *F*_(2,34)_ = 0.0507, *p* = 0.9506, *n* ≥ 11; methylcyclohexanol, *F*_(2,35)_ = 0.1433, *p* = 0.8670, *n* = 12). (E, F) Quantitative PCR analyses. RNA was extracted from *MBSw*/*dAβ* *Appl* ^*d*^/+;*MBSw*/+ and *Appl* ^*d*^/+;*MBSw*/*dAβ* fly heads. Resulting cDNA was quantified using tubulin (Tub) expression as a reference. Results shown are ratios to the reference. (E) Quantification of *dAβ* mRNA level. PCR reactions were conducted with primers specific of the *UAS-dAβ* construct (*t* test, *p* = 0.5788, *n* = 2). (F) Quantification of *Appl* mRNA level (*t* test, *p* = 0.1931, *n* = 2). Bars, Mean ± SEM. PI, Performance index. ns, not significant.

A simple hypothesis to explain how β-secretase overexpression affects memory is that it leads to an increase in Aβ production. Indeed, it has already been shown in mammals that β-secretase overexpression leads to an increase in amyloid peptides production, thus generating a gain-of-toxicity [54]. However, the lack of efficient antibodies that could specifically recognize endogenous dAβ in the MB of *Appl* ^*d*^/+;*MBSw*/*dBACE* flies did not allow us to analyze dAβ production. To circumvent this issue, we analyzed memory in transgenic flies expressing *Drosophila* amyloid peptides, a model to mimic a gain-of-toxicity context. For this purpose, we used a construct allowing expression of a putative dAβ peptide corresponding to the Aβ region of APP [29]. The expression of dAβ peptides in adult MB did not affect STM (Fig 4A), while expression in the MB of APPL partial LOF flies (*Appl* ^*d*^/+;*MBSw*/*dAβ)* resulted in STM scores significantly decreased compared to that of the controls (Fig 4B). When *Appl* ^*d*^/+;*MBSw*/*dAβ* flies were not fed with RU, they displayed a score significantly higher than that of flies of the same genotype fed with RU (Fig 4B), showing that the phenotype is specific of dAβ expression in the adult MB. We next analyzed learning and observed that *Appl* ^*d*^/+;*MBSw*/*dAβ* flies exhibited a wild-type performance (Fig 4C). We verified that dAβ expression did not affect either electric shock reactivity or aversive odors acuity (Fig 4D). Our results show that the overexpression of amyloid peptide leads to a STM deficit only when associated to an APPL partial LOF.

We next verified that the memory deficit observed in *Appl* ^*d*^/+;*MBSw*/*dAβ* was not caused by an increase in dAβ expression compared to that of *MBSw*/*dAβ* flies (Fig 4E). We also ruled out a transcriptional effect of dAβ expression on *Appl* by showing that there was no difference in *Appl* mRNA level in *Appl* ^*d*^/+;*MBSw*/*+* and *Appl* ^*d*^/+;*MBSw*/*dAβ* flies (Fig 4F). In conclusion, overexpression of either the β-secretase or dAβ peptides lead to similar phenotypes: an exacerbation of the memory deficit observed in an APPL LOF background, while learning remains unaffected.

## Discussion

Initial mechanisms responsible for the appearance of cognitive impairment in AD are still unclear. Particularly, the potential influence of APP loss-of-function and Aβ toxicity, respectively and in combination, has been poorly characterized. Here, we used *Drosophila* as a model to study the involvement of the APP pathway in memory. We investigated the incidence on memory of transient overexpression in the MB of either the β-secretase or amyloid peptides. We observed that it is deleterious for short-term memory only when associated to APPL partial loss-of-function, showing a functional interaction between APPL and amyloid peptides in memory formation.

In transgenic hAPP-mice, increased expression of human BACE1 has been shown to worsen learning and memory deficits [55,56], while in normal mice, gene knock-in of hBACE1 generated AD-relevant cognitive impairment [17]. In the present study, we thus anticipated that increasing the amyloidogenic pathway by overexpressing the fly β-secretase would have a negative impact on memory. However, although dBACE overexpression was induced for 2–3 days, STM remained unaffected. In contrast, dBACE overexpression driven in the MB of flies expressing APPL at a reduced level was deleterious for memory as it exacerbated the STM deficit due to APPL partial LOF.

Because dBACE overexpression impairs memory only in an APPL partial LOF background, the data suggest that this effect is connected to the APPL pathway. Interestingly, learning is preserved, a phenotype reminiscent of that of APPL partial LOF alone [40], giving support to the hypothesis that dBACE’s negative influence on memory is connected to the APPL pathway. We verified that dBACE expression did not modulate *Appl* transcription. Although the connection between dBACE and APPL could be indirect, a simple hypothesis is that dBACE overexpression affects memory via APPL processing. In mammalian cell cultures, increased activity of BACE1 elicits profound alterations in APP metabolism such as elevated levels of sAPPβ, CTFβ and Aβ peptides, reflecting an increase of the amyloidogenic pathway; and decreased levels of sAPPα, indicating a decrease of the non amyloidogenic pathway [57–60]. In the fly, dBACE constitutive overexpression in photoreceptor cells resulted in vacuole formation [61]. These vacuoles were comparable to the ones observed in APPL or dAβ-overexpressing flies, and may thus result from an increased production of toxic dAβ peptides when dBACE is overexpressed [29]. dBACE constitutive neuronal co-expression with APPL generates CTFβ and numerous amyloid deposits [29]. We thus hypothesized that dBACE overexpression in the adult MB led to an increase in amyloid peptide synthesis. To investigate this hypothesis, we sought to analyze directly the effect on memory of amyloid peptide overexpression.

Several studies have used *Drosophila* to model AD with constitutive human or *Drosophila* Aβ expression: neuronal expression of human Aβ42 leads to a STM deficit in young adults (5 days old), a learning deficit in older flies (14–15 days old), and formation of amyloid deposits in 48 days old flies [33,35]. Similarly, constitutive dAβ expression has been shown to generate amyloid aggregates in old flies (30 days old), whereas no gross abnormalities were seen in 2 days old flies [29]. Two days induction of Arctic Aβ42 overexpression in adult neurons has been shown to increase fly mortality and to induce neuronal dysfunction without evidence of neuronal cell loss [62]. In the present study, dAβ transient expression in the adult MB is therefore unlikely to generate neurodegeneration.

We observe that dAβ expression in adult MB neurons does not perturb STM, whereas combined with APPL loss-of-function it is deleterious for memory. This result is similar to that observed when the β-secretase is overexpressed, raising the possibility that they rely on similar mechanisms. It has been proposed that the physiological role of APP as a modulator of cell-cell and cell-substratum adhesions in neurites and synapses may be disturbed by its interaction with Aβ [63]. Direct interactions between Aβ fibrils and APP have been observed, Aβ thus acting as a ligand to its own precursor to enhance APP multimerization, a potential toxic mechanism [64–68]. A more recent study has shown that mimicking Aβ binding to APP in order to promote APP multimerization in viable neurons triggers the amyloidogenic pathway, resulting in increased BACE1 levels and Aβ production [69]. In our study, amyloid peptide exacerbates APPL loss-of-function, thus potentiating memory decline. One hypothesis is that dAβ-induced memory toxicity may result from a direct interaction between dAβ and APPL.

Altogether our data give further support to the proposal that a deficit in the normal physiological role of APP underlies the initial development of AD. Binding of Aβ fibrils to the extracellular juxtamembrane domain of APP [66–68] could trigger APP loss-of-function and increase β-secretase cleavage, a process that thus could cause a pathogenic feedback loop.
